# A mechanics model in design of flexible nozzle with multiple movable hinge boundaries

**DOI:** 10.1016/j.heliyon.2023.e12927

**Published:** 2023-01-11

**Authors:** Chengguo Yu, Zhi Li, Zhili Zhang, Licheng Meng, Guishan Wang, Yan Shi, Cunfa Gao

**Affiliations:** a Xi'an Research Institute of High Technology, Xi'an, 710025, China; b China Aerodynamics Research and Development Center, Mianyang, 621000, China; c State Key Laboratory of Mechanics and Control of Mechanical Structures, Nanjing University of Aeronautics and Astronautics, Nanjing, 210016, China

**Keywords:** Variational method, Flexible nozzle, Movable boundaries, Energy principle

## Abstract

In this paper, we propose a modified variational approach to predict the morphology of the flexible nozzle used in wind tunnel. Different from previous studies, the movements of the multiple hinges are considered as movable displacement boundary conditions during establishing the potential energy functional. The cubic spline interpolation method is employed to supply the supplementary boundary conditions in calculation of the functional minimization problem. Current analytical model is verified by experiments carried out on a fixed-flexible nozzle structure whose geometries and materials are the same as those from a commissioned supersonic nozzle. The maximum deviation between the predictions from theoretical method and laser displacement testing does not exceed 0.5 mm. This method can also deal with the large deflection beam problem with multiple movable boundaries.

## 1 Introduction

High speed wind-tunnel testing facilities play significant roles in both the fields of science and engineering [1]. They can provide a near-perfect aero-thermodynamic environment for the developments of supersonic or hypersonic aircrafts [2,3]. The core component in the facility is the nozzle part which can generate a uniform flow at the exit section with designed Mach number. Usually, the nozzle component is composed of two symmetric flat walls and two symmetric curved walls. Apparently, the flow quality is mainly determined by the morphology of the curved walls. The relationship between the wall contour and outlet flow quality is a key concern from aerodynamicists. As early as 1929, the method of characteristics/boundary-layer (MOC/BL) technique was first brought out by Prandtl and Busemann [4]. Since then, this approach has been the basic guidelines for nozzle design with inviscid flow assumption [5,6]. Actually, this method decouples the influences between the mainstream and the boundary layer flows, which is suitable for the scenarios in low-Mach-number wind tunnels. For high-Mach-number cases, the boundary layer becomes thicker, and its influences on the characteristics of the mainstream flow should be carefully inspected [7]. With the developments of computational fluid dynamics (CFD) and the computer clusters, the Navier-Stokes equation based solvers are widely employed to calculate the coupling effects of the boundary flow and core flow [8,9]. As a result, the Mach number deviations between design prediction and real object are dramatically reduced [10].

**Fig. 1:**
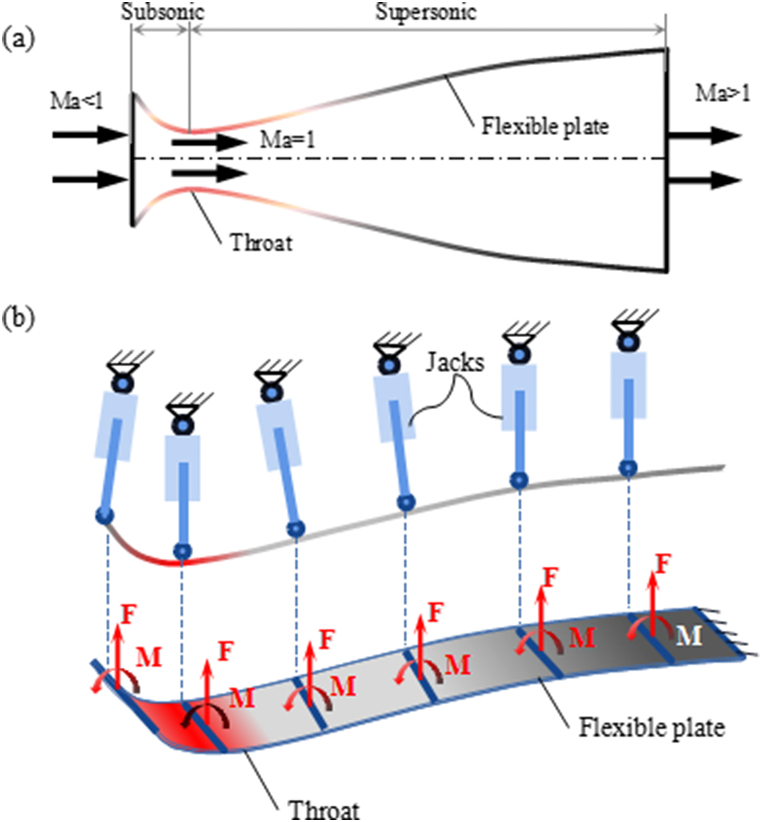
Schematic illustrations of (a) the fixed-flexible nozzle contour and (b) its operating principle.

After nearly a century of development, the aerodynamicists have founded a series of mature approaches to design nozzle contours for high-Mach number core flows [11,12]. When the sight turned to the engineering reality, a new problem appeared. Based on the CFD calculation, a demanded Mach number flow is corresponding to a fixed nozzle contour. Thus, in order to test the aero-thermodynamic properties at different Mach numbers, a series of fixed-block nozzles should be constructed, which caused waste in space and resources. The concepts of fixed-flexible nozzle [13] as well as full flexible nozzle were brought out. Usually, a flexible nozzle consists of fixed flat wall, flexible walls, planar motion mechanisms and hinge connections. Fig. 1(a) shows a typical full flexible nozzle mechanism used in supersonic wind tunnel. The flexible wall is hinged to six jacks which can realize sliding and rotating motion at the same plane as shown in Fig. 1(b). After driven by the hydraulic system, a given elongation or shortening is applied to each jack rod. After bending deformation of the flexible plates and rotations of the jacks, a new equilibrium condition will achieve. Guaranteed by minimization potential principle, a determinate contour is corresponding to the jack elongations or shortenings.

The reconstructions of the flexible plates after deformation become the key issue during design of flexible nozzles. Researchers and engineers have developed several approaches to calculate the deformed contours of the flexible plates. For the single-jack nozzle [14], which is usually simplified as a cantilever beam, the elliptic integral is an efficient tool to calculate its deflection [15,16]. It is notable that, elliptic integral method can predict both the displacements of the beam and hinge point. In addition, shooting method [[17], [18], [19]], finite difference method [20,21], and finite element method (FEM) are also introduced to deal with the large deformation beam problem. However, for the case with multiple jacks, it is difficult to determine the limits of the integration or element boundaries due to the hinge point movements. With the development of computer technology, commercial FEM software such as ABAQUS or ANSYS supplies a powerful instrument for dealing with the reconstruction problems. However, as known to all, the modelling process consumes a large amount of time and labors, which is seriously inconvenient at design stage due to geometry variations.

For traditional problems of large deformation of elastic beam [22], the loads are not complicated, e.g., dead load [23] and follower load [24]. The former one is constant in magnitude and direction [25,26]. The latter one is variational followed with deformed beam, generally the magnitude is unchanged but the direction keeps consistent with beam during deformation [27]. In compliant mechanisms [28], the load is replaced by rigid rod motion without elongation and shorting, in which the beam and rod stay clamped. Here, we consider the rod elongation and rotation in large deformation of beam, and the load direction also needs to be solved in this mechanics model. Particularly, it is a multiple boundaries problem under the actions of several rods. Reexamine current approaches and their limitations. The multiple movable boundaries seem to be the key issues. Here, we re-derived the potential energy functional of the mechanism system with movable hinges treated as displacement boundaries. The negligible axial elongation assumption is also introduced. Lagrange multipliers are employed to release all displacement constraints for the functional extreme value problem. The derivations and solutions to this large deformation beam problem with multiple movable hinge boundaries are presented in Section 2. In Section 3, the finite element method is employed to examine the validity of the theoretical model. In Section 4, the theoretical model is further verified on an experiment platform molding from a commissioned supersonic flexible nozzle. At last, the work is concluded in Section 5.

## 2 Theoretical model

### 2.1 Potential energy functional

**Fig. 2:**
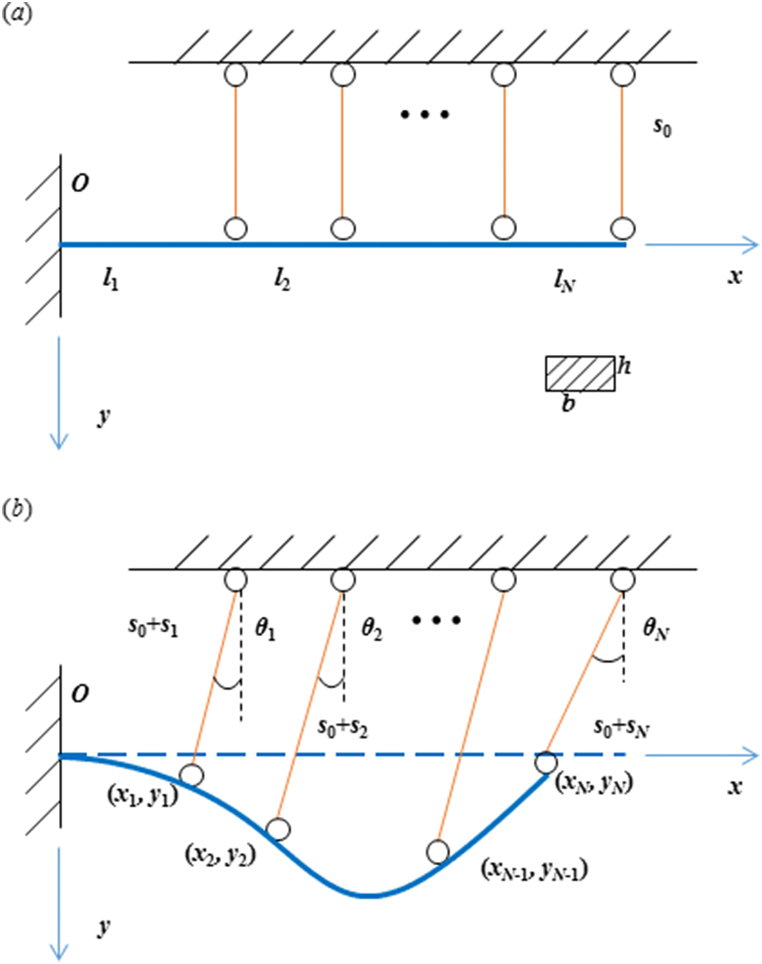
A simplified mechanics model for a flexible beam with multiple movable hinge connections. (a) The initial state without jack movements and (b) the deformation of the flexible plate after jack stimulations.

As shown in Fig. 2(a), by geometric symmetry, the flexible nozzle system is simplified as a straight cantilever hinged to multiple rigid jacks. After driven by the elongation or shortening of each jack, the beam deforms to a curved contour and the jacks rotate with the fixed hinges as shown in Fig. 2(b). Apparently, the elongation or shortening of each jack can be treated as known values. We introduce an assumption that the axial elongation of the beam section between adjacent hinges is negligible, which can be treated as constraint conditions in functional extreme problem. Thus, we can construct the potential energy of the structures with Lagrange multipliers as [29],

(1) $$\Pi =\int_{0}^{x_{N}}\frac{1}{2}EI{\left[\frac{w^{\prime \prime }}{{\left(1+w\prime^{2}\right)}^{\frac{3}{2}}}\right]}^{2}dx+\sum_{i=1}^{N}\lambda_{i}\left(\int_{x_{i-1}}^{xi}\sqrt{1+w\prime^{2}}dx-l_{i}\right)$$

where, Π represents the total potentials of system. *E* and *I* are the Young's modulus and inertia moment, respectively. *w* denotes the deflection, i.e., the contour of the flexible plate. $\lambda_{i}$ (*i* = 1, 2, …, *N*) are Lagrange multipliers, which can be determined by one order variation of Π. *l*_*i*_ denotes the length of *i*^th^ beam section. The superscript “ ’ ” denotes the differential operator. The minimization of potentials leads to *δ*Π = 0, i.e.

(2) $$\begin{array}{l} EI\sum_{i=1}^{N}\left[\int_{x_{i-1}}^{x_{i}}\left(\frac{3w^{\prime }{\left(w^{\prime \prime }\right)}^{2}}{{\left[1+{\left(w^{\prime }\right)}^{2}\right]}^{4}}-\frac{w^{\prime \prime \prime }}{{\left[1+{\left(w^{\prime }\right)}^{2}\right]}^{3}}+\frac{\lambda_{i}}{EI}\frac{w^{\prime }}{\sqrt{1+{\left(w^{\prime }\right)}^{2}}}\right)\delta w^{\prime }dx+\delta \lambda_{i}\left(\int_{x_{i-1}}^{x_{i}}\sqrt{1+{\left(w^{\prime }\right)}^{2}}dx-l_{i}\right)\right]=0\\ \left(i=1,2,\ldots ,N\right) \end{array}$$

The Euler-Lagrange equations and constraints are obtained as

(3) $$\frac{3w^{\prime }{\left(w^{\prime \prime }\right)}^{2}}{{\left[1+{\left(w^{\prime }\right)}^{2}\right]}^{4}}-\frac{w^{\prime \prime \prime }}{{\left[1+{\left(w^{\prime }\right)}^{2}\right]}^{3}}+\frac{\lambda_{i}}{EI}\frac{w^{\prime }}{\sqrt{1+{\left(w^{\prime }\right)}^{2}}}=0\;\left(i=1,2,\ldots ,N\right)$$

(4) $$\int_{x_{i-1}}^{x_{i}}\sqrt{1+{\left(w^{\prime }\right)}^{2}}dx-l_{i}=0\;\left(i=1,2,\ldots ,N\right)$$

Actually, Eq. (3) is not strictly correct, because the summation of the bending energy is neglected. This treatment breaks the entity of the beam, which is a common problem faced by most of the segmental methods. In current work, we will bring in extra boundary conditions to fix this problem.

### 2.2 Solutions

By approximation of Taylor's expansion, Eq. (3) can be re-written as

(5) $$\begin{array}{l} w=w\left(x_{0}\right)+w^{\prime }\left(x_{0}\right)\left(x-x_{0}\right)+\frac{w^{\prime \prime }\left(x_{0}\right)}{2!}{\left(x-x_{0}\right)}^{2}+\\ \;\frac{w^{\prime \prime \prime }\left(x_{0}\right)}{3!}{\left(x-x_{0}\right)}^{3}+\frac{w^{\left(4\right)}\left(x_{0}\right)}{4!}{\left(x-x_{0}\right)}^{4}+\cdots \end{array}$$

where, the deflection and its first-order derivative at the left end of *i*th beam can be determined by the fix boundary or the right end of the (*i* − 1)^th^ beam. The third and higher order derivatives can be expressed by derivatives of the first two orders as following

(6) $$\left\{ \begin{array}{l} w^{\prime \prime \prime }=\frac{3w^{\prime }{\left(w^{\prime \prime }\right)}^{2}}{1+{\left(w^{\prime }\right)}^{2}}+\frac{\lambda }{EI}w\prime {\left[1+{\left(w^{\prime }\right)}^{2}\right]}^{\frac{5}{2}}\\ w^{\left(4\right)}=\frac{3{\left(w^{\prime \prime }\right)}^{3}\left[1+5{\left(w^{\prime }\right)}^{2}\right]}{{\left[1+{\left(w^{\prime }\right)}^{2}\right]}^{2}}+\frac{\lambda }{EI}w^{\prime \prime }{\left[1+{\left(w^{\prime }\right)}^{2}\right]}^{\frac{1}{2}}\left[12{\left(w^{\prime }\right)}^{4}+13{\left(w^{\prime }\right)}^{2}+1\right]\\ w^{\left(5\right)}=\frac{15w^{\prime }{\left(w^{\prime \prime }\right)}^{4}\left[7{\left(w^{\prime }\right)}^{2}+3\right]}{{\left[1+{\left(w^{\prime }\right)}^{2}\right]}^{3}}+\frac{\lambda }{EI}\frac{3w^{\prime }{\left(w^{\prime \prime }\right)}^{2}}{{\left[1+{\left(w^{\prime }\right)}^{2}\right]}^{\frac{1}{2}}}\left[47{\left(w^{\prime }\right)}^{4}+60{\left(w^{\prime }\right)}^{2}+13\right]\\ +\frac{\lambda^{2}}{{\left(EI\right)}^{2}}w^{\prime }\left[12{\left(w^{\prime }\right)}^{2}+1\right]{\left[{\left(w^{\prime }\right)}^{2}+1\right]}^{4}\\ \cdots \end{array}\right.$$

Now, substituting Eq. (6) into Eq. (5), the unknowns are Lagrange multipliers and second-order derivative of the deflection. Notice that, the hinge boundaries are

(7) $$\left\{ \begin{array}{l} x_{i}=\sum_{n=1}^{i}l_{n}-s_{i}\;\sin\;\theta \\ y_{i}=s_{i}\;\cos\;\theta -s_{0}\\ w\left(x_{i}\right)=y_{i}\\ w^{\prime }\left(x_{i}\right)=\alpha_{i} \end{array}\right.\left(i=1,2,\ldots ,N\right)$$

where, (*x*_*i*_, *y*_*i*_) are coordinates of each hinge point at deformed configurations. *s*_0_ and *s*_*i*_ represent the jack length before and after loading, respectively. Here, we introduced an extra boundary constraint *α*_*i*_, which denotes the slope of the beam at the hinge point. Actually, the slope boundaries are used to eliminate the errors caused by segmental integrations in Eqs. (2) and (3). As verified by simulations, when the jack elongation or shortening is within 50% of its original length, the slopes can be approximated by spline interpolation by neglecting their rotations. After instituting the slope boundaries into Eq. (7), the rotation of the jacks can be derived by Eqs. (3) and (4). It is notable that, the method of spline interpolation only supplies the initial reference slopes of the beam at every nodes, which does not truly fix the rotation of the jacks.

## 3 Simulation examples

**Fig. 3:**
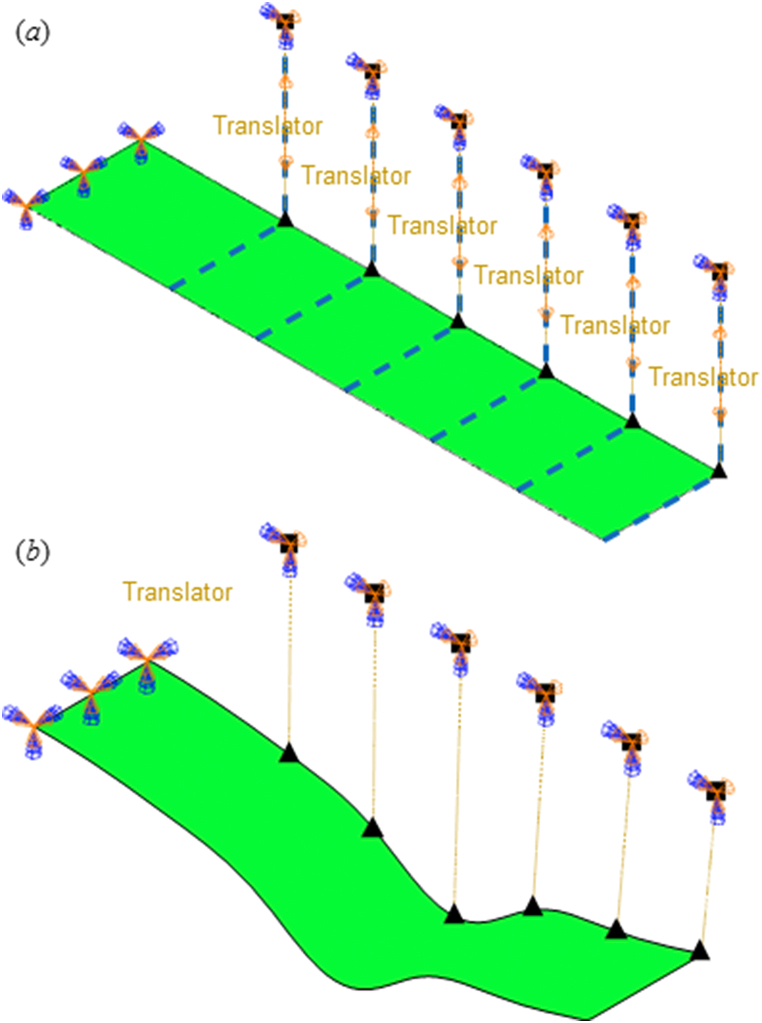
The finite element models of the flexible plate with six jacks. (a) A flexible shell structure with six “translator” kinematic pairs. (b) A steady state configuration of the flexible shell after jacks stimulations.

In this section, the commercial software ABAQUS is employed to simulate the cantilever beam with multiple hinge boundaries as shown in Fig. 3(a). The thickness of flexible nozzle is much smaller than the other two dimensions, which satisfies the shell assumptions. In finite element analyses (FEA), four-node shell element with reduced integration is selected to model the cantilever in order to improve calculation precision and efficiency. The fragment length is set as 250 mm (mm) for the first section (*l*_1_) and 150 mm for the other five sections (*l*_2_-*l*_6_), respectively. The width and thickness of the plate are 200 mm and 5 mm, respectively. The original length of each jack is 250 mm. The jacks are simulated by Connector Element with translational kinematic pairs along their axial direction and planar revolute pairs at hinge points, respectively. After stimulation by the jacks, the flexible plate will achieve a steady state with a determinate contour as shown in Fig. 3(b).

The shell element is assigned with steel and its elastic modulus (*E*) and Poisson's ratio (ν) are assigned to 210 GPa and 0.3, respectively. The jack axial displacement is defined on the local coordinate system with the central axis assigned as local *x* axis for each jack. A “translator” kinematic pair is assigned to each jack, where only sliding movement is allowed in local coordinates. And convergence of mesh sizes was tested to ensure computational accuracy.

**Fig. 4:**
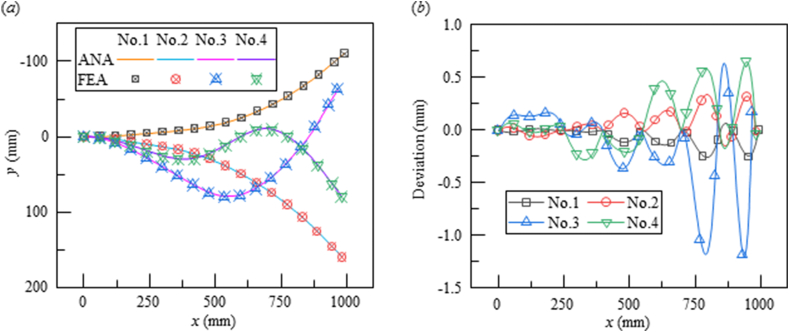
Predictions of the plate deflections from analytical analyses (ANA) in solid line and finite element analyses (FEA) in symbols, respectively. (a) Deflections and (b) deviations of the contours predicted from ANA and FEA in the four configurations, respectively. The square, circle, upper triangle and lower triangle symbols representing the deformations stimulated by the jack movements form No.1 to No.4 in Table 1, respectively.

Fig. 4 demonstrates the deformed configuration of the flexible plate subjected to multiple displacement loadings as shown in Table 1. As depicted in Fig. 4(a), the solid lines and symbols represent the contours predicted from theoretical model and FEA, respectively. Different from existing analytical model, the current model can predict the movements of the hinge points with high accuracy. The average deviation of hinge points does not exceed 0.08 mm in all cases, which is approximately 0.1% divided by the displacement loading or 0.06% divided by the length of the segmental beam. For the contour line, the maximum deviation appears in No. 3 in Table 1 with a value of 1.2 mm as exhibited in Fig. 4(b). In other three cases, the maximum deviations do not exceed 0.6 mm. According to Eq. (5), only the first four items of Taylor polynomial is adapted to fit the deflection contours, which means this prediction can not accurately describe the contours with drastic curvature changes as shown in No. 3 and No. 4 Re-examine the deviations of the above model (in Fig. 4(b)), the relatively large deviation always come out at the slope transformation section, which is uncommon in actual situations due to the unwished stress concentrations at these cases. Actually, the real curvature variations in flexible nozzle are always smooth and monotonic. The current simplifications can meet the accuracy requirements completely.

**Table 1:** The stimulation movement of each jack in the four cases.

Jack number		1	2	3	4	5	6
*x* coordinate (mm)		250	400	550	700	850	1000
Stimulation combination (mm)	No. 1	−5	−10	−20	−40	−70	−100
	No. 2	10	20	40	70	110	160
	No. 3	30	60	80	60	10	−60
	No. 4	20	30	10	−10	20	80

## 4 Experiments

### 4.1 Experiment verification

**Fig. 5:**
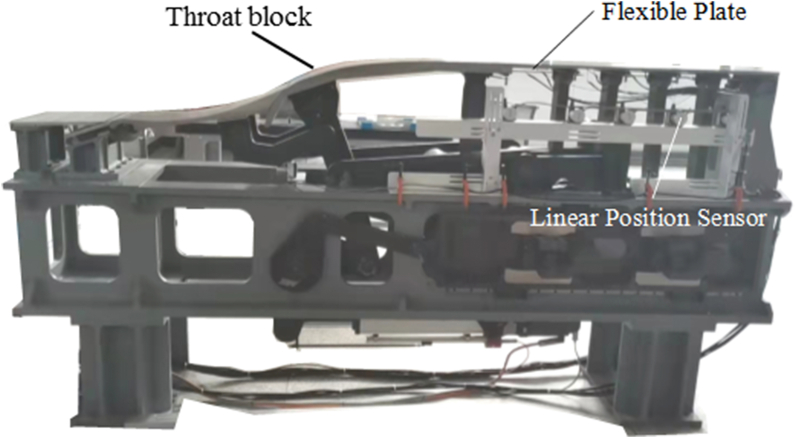
A testing platform molding from a commissioned supersonic nozzle in China Aerodynamics Research and Development Center.

Fig. 5 shows a fixed-flexible nozzle facility molding from a commissioned 0.3 m supersonic wind-tunnel in China Aerodynamics Research and Development Center, Mianyang, China. It can achieve stable output of fluid in six Mach numbers including 1.0, 1.3, 1.5, 2.0, 2.5 and 3.0. In order to verify our theoretical model, some modifications were made to satisfy the displacement boundary conditions. As shown in Fig. 5, six linear position sensors were installed to simulate the jack movements. And their virtual elongations or shortenings can be easily read from the electrical signals. Although the platform is driven by crankshaft mechanism, we can assume it is driven by the virtual jacks. Firstly, a laser displacement system was employed to draw the plate contour point by point. Six nozzle contours corresponding to flows of Mach number ranging from 1.0 to 3.0 were measured as standard references, respectively. Meanwhile, the displacement at each virtual jack was recorded by the linear position sensors. After substituted into the theoretical model in Section 2, the predicted contours were obtained.

**Fig. 6:**
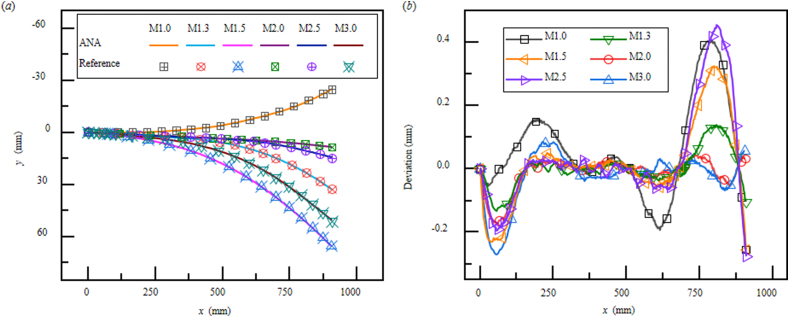
Predictions of the plate deflections from analytical analyses (ANA) in solid line and reference contours in symbols, respectively. (a) Deflections and (b) deviations of the contours from ANA and laser displacement testing (reference contours), respectively. The square, circle, and triangle symbols representing the reference contours for Mach number 1.0 to 3.0.

As shown in Fig. 6(a), the theoretical model can predict the plate contours of the true supersonic nozzle with high accuracy. The reference plate contours are the true profile curves used in a supersonic wind-tunnel on active duty. The deviations between analytical model and laser sensor testing do not exceed 0.5 mm (in Fig. 6(b)), which satisfy the design requirements. In other words, current model can serve as guidelines in design of supersonic nozzles.

### 4.2 A simple application

**Fig. 7:**
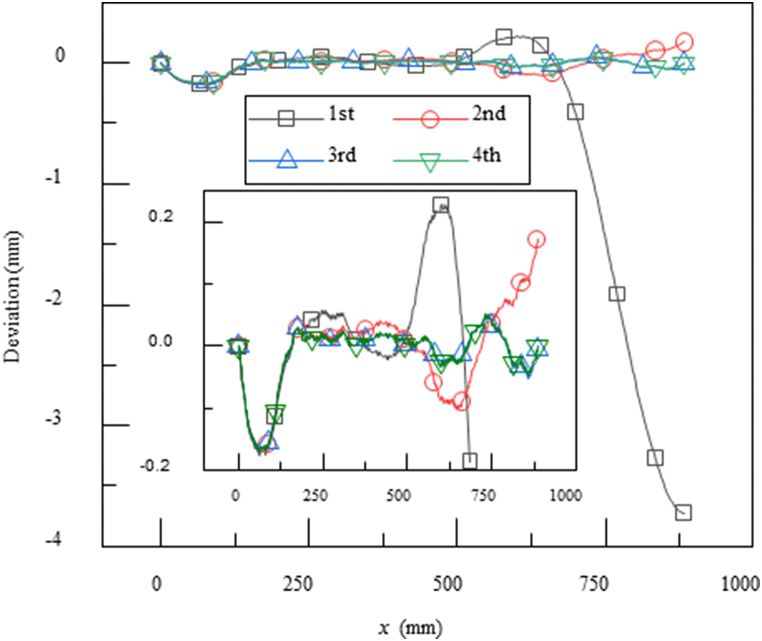
Deviations between calculations from analytical model and reference contour after one to four iterations, respectively.

The current theoretical model offers an efficient strategy for the mechanical design of the nozzle facility. For example, when the nozzle contour for a flow with a certain Mach number has been obtained by CFD, how can we design the lengths, locations and driven displacements of the jacks? Here, a simple inverse problem of the jackleg elongations is presented. Take the nozzle contour of Mach number 2.0 for example (in Fig. 6(a)). Firstly, we get the horizontal coordinates of the hinge points and find their vertical coordinates in the plate form reference contour. The vertical coordinates are set as jack elongations (or shortenings) in theoretical model. In the first iteration, the deviations from reference contour are shown in Fig. 7 with square symbol. Then, a new set of jack elongations are obtained by subtracting the deviation at each hinge point, which are the input for the second iteration. The third and higher order iterations are carried out in the similar way. As shown in Fig. 7, after three iterations, the contour deviations between theoretical model and references are within acceptable limits. The jack rotations, hinge coordinates and reaction forces are calculated accordingly.

## 5 Conclusions

In the current work, a modified variational method is established to predict the deflections of a flexible plate with multiple movable hinge boundaries. As verified by finite element methods and experiments, the theoretical model has high accuracies in calculations of plate deflection as well as hinge support movements. As inspired by supersonic flexible nozzle facility, this method can serve as a feasible tool in design of supersonic nozzle in contour prediction, hinge point location and kinematic relation designs. Furthermore, this method also has potentials in design of flexible motion mechanism such as morphing wing aircrafts.

## Author contribution statement

Chengguo Yu: Conceived and designed the experiments.

Zhi Li; Licheng Meng: Performed the experiments.

Zhili Zhang; Guishan Wang: Contributed reagents, materials, analysis tools or data.

Yan Shi: Analyzed and interpreted the data; Wrote the paper.

Cunfa Gao: Analyzed and interpreted the data.

## Funding statement

Prof. Yan Shi was supported by 10.13039/501100001809National Natural Science Foundation of China [12072150], Have we correctly interpreted the following funding source(s) and country names you cited in your article: Innovative Research Group Project of the National Natural Science Foundation of China, China; Joint Fund of Advanced Aerospace Manufacturing Technology Research; State Key Laboratory of Mechanics and Control of Mechanical Structures, China? [U1937601], Research Fund of 10.13039/501100011402State Key Laboratory of Mechanics and Control of Mechanical Structures [MCMS-I-0221Y01].

Cunfa Gao was supported by 10.13039/100014718Innovative Research Group Project of the National Natural Science Foundation of China [51921003].

## Data availability statement

Data will be made available on request.

## Declaration of interest's statement

The authors declare no competing interests.
